# The economic burden of hemodialysis and associated factors among patients in private and public health facilities: a cross-sectional study in Addis Ababa, Ethiopia

**DOI:** 10.1186/s12962-024-00530-7

**Published:** 2024-04-04

**Authors:** Tiruneh Amsalu Baye, Hamelmal Gebeyehu, Mahteme Bekele, Semira Abdelmenan, Tigistu Adamu Ashengo, Berhanu Mengistu

**Affiliations:** 1 Addis Continental Institute of Public Health, Addis Ababa, Ethiopia; 2 Department of Internal Medicine, St. Paul’s Hospital Millennium Medical College, Addis Ababa, Ethiopia; 3 Department of Surgery, St. Paul’s Hospital Millennium Medical College, Addis Ababa, Ethiopia; 4 Jhpiego, an affiliate of the Johns Hopkins University, 21231 Baltimore, MD USA; 5 Department of Human Nutrition, Institute of Public Health, University of Gondar, Gondar, Ethiopia

**Keywords:** Renal disease, Hemodialysis, Direct cost, Indirect cost, Ethiopia

## Abstract

**Introduction:**

The treatment of kidney disease, including hemodialysis, poses challenges in healthcare and finances. Despite limited data on hemodialysis costs and determinants in Ethiopia, existing literature indicates a paucity of evidence regarding the economic burden of hemodialysis. This study aims to evaluate the direct and indirect costs of hemodialysis among end-stage renal disease (ESRD) patients, alongside associated factors, among selected governmental and private institutions in Addis Ababa, Ethiopia.

**Methods:**

An institutional-based cross-sectional study using a simple random sampling technique was conducted from September 10 to November 1, 2021. One hundred twenty-eight patients participated in the study. Data was collected using an interviewer-administered questionnaire. The analysis used proportion and frequency measures of central tendency and linear regression measures. Both simple and multiple linear regression models were used to assess associated factors. The final model used a P value < 0.05 at 95% confidence interval (CI) was used to determine significance.

**Result:**

The mean cost of hemodialysis in a representative sample of selected hospitals in Addis Ababa was 7,739.17 $ ±2,833.51 $, with direct medical cost contributing 72.9% of the total cost. Furthermore, the institution type (private or public) and duration on hemodialysis were associated with an increased cost of hemodialysis.

**Conclusion:**

Our findings underline the necessity for policymakers, program administrators, and healthcare institution executives to prioritize this group, recognizing the substantial load they bear and extending these services in government facilities to a broader patient population.

## Plain language summary

**What is known?:**

Chronic kidney disease is the leading cause of sickness and death, affecting an estimated 10% of the population in 2015. Treatment of Kidney disease, including hemodialysis, presents not solely a medical concern but also a financial aspect. Therefore, we tried to assess the direct and indirect cost of hemodialysis among chronic kidney disease patients and associated factors among selected government and private institutions.

**What did we do?:**

The study’s objective was to evaluate the direct and indirect costs of hemodialysis in patients with chronic kidney disease and examine the associated factors within selected government and private institutions. We selected the institutions after expert consultation due to their high patient flow. An institution-based cross-sectional study was conducted, using an interviewer administered semi structured-questionnaire.

**What did we find?:**

We found the mean cost of hemodialysis in a representative sample of selected hospitals in Addis Ababa to be 7,739.17$ ±2,833.51$, with direct medical cost contributing 72.9% of the total cost. Furthermore, the institution type (Private or Public) and number of years on hemodialysis were predictors of increased cost. Moreover, our findings have highlighted various strategies employed by patients facing challenges covering these expenses. Most patients resort to seeking assistance from family and friends, reducing the frequency of hemodialysis sessions, and cutting back on prescribed medications. It is important to note that several coping mechanisms can adversely affect patients’ health, given that they involve skipping crucial life-saving treatments.

**What do the results mean?:**

We found out that the cost of hemodialysis was relatively high among the study participants. Therefore, policymakers, programmers, health institution leaders should pay closer attention to these patients as they face significant health and financial burdens.

## Introduction

Chronic kidney disease (CKD) is a leading cause of morbidity and mortality in developed and emerging countries, affecting an estimated 10% of the global population in 2017 [1, 2]. CKD has reached a concerning proportion worldwide, and by 2030 it is estimated that more than 70% of patients with end-stage renal disease (ESRD) will be from countries with comparable demographics to those of Sub-Saharan Africa [3, 4]. Hemodialysis is extracting extra water, solutes, and pollutants from the blood of patients whose kidneys are no longer perfectly functional [5]. Aside from treatment-related challenges, there are significant financial challenges in managing chronic kidney diseases [6].

The economic burden of hemodialysis is well documented globally. For instance, the annual healthcare spending on hemodialysis in China is approximately fifty billion dollars [7]. African countries have also reported significant health care expenses associated with hemodialysis; in South Africa and Tanzania, the estimated annual cost for hemodialysis was $31,993.12 and $27,440, respectively [8, 9]. Moreover, a report from Sudan has also revealed the estimated cost for hemodialysis was $6,847 annually [10, 11]. A previous study conducted at governmentally owned hemodialysis centers in Ethiopia reported $4,466.59 annual cost of hemodialysis [12].

Various studies have revealed that socio-demographic factors like age [12–15], sex [13], and wealth status [13] displayed significant association with increased expenses related to hemodialysis. Similarly, conditions such as anemia [13], diabetes [12], and heart failure [12] were associated with elevated costs of hemodialysis.

Ethiopia spends just 5% of its gross domestic product (GDP) on healthcare and is a significant recipient of international assistance, primarilyused to fund communicable disease control and government-run primary care. Until recently, renal replacement therapies were funded by sporadic charitable donations [16]. While hemodialysis services are now being offered in several government-owned secondary and tertiary care institutions, the delivery of these services could be better [17]. The limitations of hemodialysis services stem from insufficient machines, lack of reagents, and the complex nature of the illness [17]. Consequently, patients who cannot afford private care, which costs significantly more, are waitlisted for this life time treatment.

Despite the increasing prevalence of chronic kidney disease in Ethiopia, there is a paucity of data on the cost of hemodialysis and associated factors in public and private health facilities. Therefore, this study aimed to ascertain the cost of hemodialysis and associated factors of patients attending treatment in public and private facilities. In addition, the study looked at the different coping mechanisms employed by those patients who could not afford hemodialysis.

## Methods and materials

### Study design and period

An institution based cross-sectional study design was employed from September 10 to November 1, 2021. The study was conducted in selected government and private institutions in Addis Ababa. The selected institutions include St. Paul’s hospital millennium medical college, Zewditu Memorial hospital, Menelik hospital, Hayat hospital and Ethio-Tebib hospital. St. Paul’s hospital Millennium medical college is a pioneer in modern hemodialysis in Ethiopia and the only transplant center in the country. Zewditu Memorial Hospital and Menelik Hospital are among the hospitals under the Addis Ababa health bureau to provide hemodialysis service. In contrast, Hayat hospital medical college and Ethio Tebib are privately owned hemodialysis centers. Considering their hemodialysis services and significant patient caseload, these institutions were chosen in consultation with nephrologists.

### Study participants

The sample size was calculated using the single population mean formula, $4466.59 estimated cost of hemodialysis at a 5% standard of error [12]. Finally, after adding a 10% non-response rate 128 patients were required for this study. The final sample was proportionally distributed to the selected hemodialysis centers in Addis Ababa. Finally, the study participants were recruited using simple random sampling; andthe sampling frame was obtained at the selected hospitals. Those patients who were critically ill and had no attendants at the time of data collection were excluded from the study.

### Data collection

An interviewer administered semi-structured questionnaire was adapted from a previous study done in Ethiopia [12]. The questionnaire consisted of inquiries designed to evaluate the socio-demographic, clinical, and cost-related traits of the participants under study. Information about clinical aspects was extracted from the patient’s medical records.

The dependent variable is the cost of hemodialysis measured as the sum of the direct medical cost, direct non-medical cost, and indirect cost. The direct medical costs were hemodialysis sessions, drugs, lab investigations, and other related medical expenses. The direct non-medical costs included transportation, c food, and other costs that were directly related to nonmedical costs incurred by patients and their relatives. In contrast, the indirect costs include lost wages or other loss of production that impact the patients’ income.

The wealth status of the participant was assessed through the household wealth index and principal component analysis and ranked into three (Low, Medium and High) levels.

First, the questionnaire was prepared in English and translated to Amharic (local language) by subject matter experts and language experts for the purpose of checking its consistency, the text was translated back into English. The questionnaire was pretested at Tom hemodialysis center, Addis Ababa, Ethiopia using 10% of the final sample size. Five data collectors (B.Sc. MD) and two supervisors (B.Sc. MD) were recruited. Two days of training on the study’s purpose, data collection procedures, Kobo toolbox data collection tool, and ethical issue was provided. Throughout the data collection process, data collectors received feedback at two levels. First, the on-site data collection supervisor provided feedback during the data collection phase. Additionally, the principal investigator offered feedback during the daily debrief call, conducted virtually.

### Statistical analysis

The data was collected using the Kobo tool box software. The cleaned data was exported to STATA 15 software for analysis. Descriptive statistics such as frequency, percentage, mean, and Standard Deviation (SD) were used to summarize the data. A linear regression model was used to determine the association between the dependent and independent variables.

Before running the linear regression, assumptions like linearity, normality, multicollinearity (VIF (variance inflation factor) < 5), homoscedasticity and outliers, were checked and those variables that did not meet the assumptions were removed. After this,a simple linear regression analysis was conducted to see the linear association between hemodialysis cost and each of the independent variables. Variables with p value < 0.2 in the simple linear regression were considered for the final model. In the final model, variables with p-value < 0.05 were considered independent predictors of hemodialysis cost.

## Result

### Socio-demographic and economic characteristics of respondents

All the study participants had consented to participate in the study (100% response rate). Concerning the patient’s placement, the majority (56.3%) were from public institutions. Two-thirds (66.41%) of the respondents were male. The mean age of the respondents in years was 41.67 ± 13.32 (SD). As to marital status, more than half (54%) were married.

The majority (90%) of the respondents have completed their education at or above the secondary education level. Most responders were unemployed or not engaged in economic activities, with more than half belonging to this group. The mean family size being 4.45 ± 2.40SD. All but two of the respondents resided in the urban areas. Regarding wealth status, the participants are almost equally divided to low, medium and high income with percentages of 32.03%, 34.38% and 33.59% respectively (Table 1).

**Table 1:** Socio-demographic and economic characteristics of participants at selected institution in Addis Ababa, Ethiopia, 2021 (*n* = 128)

Variables	Categories	Frequency (n)	Percentage (%)
Institution	Public	72	56.25
	Private	56	43.75
Sex	Male	85	66.41
	Female	43	33.59
Age (Years)	18–35	52	40.63
	36–55	54	42.19
	56–65	17	13.28
	> 65	5	3.91
Marital Status	Married	70	54.69
	Unmarried	58	45.31
Educational Status	No Education	5	3.91
	Primary	7	5.47
	Secondary	75	58.59
	College and above	41	32.03
Occupational Status	Employed	52	40.63
	Unemployed	76	59.38
Family Size	≤ 4	77	60.16
	> 4	51	39.84
Place of Residence	Urban	126	98.44
	Rural	2	1.56
Wealth Index	Low	42	33.33
	Medium	41	32.54
	High	43	34.13

### Health service-related characteristics of respondents

More than half (55.47%) of participants stated that the health facility was more than 9 km from their residence. Regarding transportation, three fourth (75%) of the participants utilized public transportation (Table 2).

**Table 2:** Health service related characteristics of participants at selected institution in Addis Ababa, Ethiopia, 2021 (*n* = 128)

Variables	Categories	Frequency (n)	Percentage (%)
Distance (km)	<3	6	4.69
	3–6	24	18.75
	6–9	27	21.09
	>9	71	55.47
Type of transportation	Walked	3	2.34
	Public Transport	96	75.00
	Private Car	29	22.66

### Clinical characteristics and treatment modalities

Two fifth (41.41%) of the participants had been on hemodialysis for over three years, and more than two third (68%) of the participants attended hemodialysis thrice a week. The vast majority of the participants (86.72%) utilized a fistula for vascular access. Almost all the participants had another comorbidity in addition to CKD. The three most prevalent comorbidities were hypertension, anemia and chronic kidney disease mineral bone disease (CKDMBD) with a prevalence of 85.83%, 82.5% and 40%, respectively. Regarding comorbidity, 93.8% of the participants were on treatment for these conditions, other than hemodialysis (Table 3).

**Table 3:** Clinical characteristics and treatment modality of participant at selected institution in Addis Ababa, Ethiopia, 2021 (*n* = 128)

Variables	Categories	Frequency (n)	Percentage (%)
Duration hemodialysis (years)	< 1	22	17.19
	1–3	53	41.41
	> 3	53	41.41
Hemodialysis per week	Two	40	31.25
	Three	88	68.75
Vascular access	Fistula	111	86.72
	Catheter	11	8.59
	Graft	6	4.69
Presence of comorbidity	No Comorbidity	9	7.03
	Single Comorbidity	13	10.16
	Multiple comorbidities	106	82.81
Type of comorbidity	Anemia	99	82.50
	Hypertension	103	85.83
	CKDMBD	48	40.00
	Vitamin D deficiency	12	10.00
	Diabetes	22	18.33
	CVD	18	15.00
Medication other than hemodialysis	No Medication	8	6.3
	Single Medication	6	4.7
	Multiple Medications	114	89.1

### Cost of hemodialysis

The mean annual cost for hemodialysis for study participants was 7,739.17 $ (364,515.10 ETB) ± 2,833.51 $ (133,458.09 ETB) (1 USD = 47.10 ETB, 2021 fiscal year). The direct medical cost amounted to 5652.52$ ± 2,521$, comprising the largest share (72.9%) of the total cost. In comparison, the indirect cost and direct non-medical cost amounted to an average of 1,301.02 ± 1,146.68 $ (16.81%) and 736.31 ± 734.73 $ (9.51%), respectively (Table 4).

**Table 4:** Direct medical, direct non-medical, indirect and total cost of hemodialysis at selected institution in Addis Ababa, Ethiopia, 2021 (*n* = 128)

Variables	Mean ± SD of the cost (ETB)	Mean ± SD of the cost (USD)
Direct medical Cost	266,457.19 ± 118,817.03	5,646.52 ± 2,521.05
Direct non-medical cost	34,905.06 ± 34,566.38	736.31 ± 734.73
Indirect cost	63,152.85 ± 55,485.55	1,301.02 ± 1,146.68
Total Cost	364,515.10 ± 133,458.09	7,739.17 ± 2,833.51

Regarding the direct hemodialysis costs, more than half (54.6%) of participants have received a fee waiver for the incurred cost, almost two fifth (38.3%) covered the cost by themselves, and asmall proportion (7.03%) of participants covered it through a third-party mechanism such as work insurance. Almost two-thirds (64.06%) of respondents buy their medication from government owned drug stores, and more than three quarter (76.6%) bear this cost themselves. Many participants covered lab-related costs (54.69%), while a quarter (25%) had a fee waiver. The direct non-medical costs were entirely covered by the patients themselves. Almost two-thirds (65.63%) of the participants have visited other facilities in the past year aside from their primary hemodialysis center. Furthermore, the vast majority (95.3%) of the participants stated that their income was insufficient to cover the hemodialysis cost (Table 5).

**Table 5:** Further description of cost of hemodialysis at selected institution in Addis Ababa, Ethiopia, 2021 (*n* = 128)

Variables	Categories	Frequency (n)	Percentage (%)	
Payment mechanism (hemodialysis)	Self	49	38.28	
	Third party	9	7.03	
	Fee waivers	70	54.69	
Source of medication	Government drug stores	82	64.06	
	Private drug stores	18	14.06	
	Both government and private drug stores	28	21.88	
Payment mechanism (medication)	Self	98	76.56	
	Third party	27	21.09	
	Fee waivers	3	2.34	
Payment mechanism (lab investigation)	Self	70	54.69	
	Third party	26	20.31	
	Fee waivers	32	25.00	
Visited other health facility	Yes	44	34.38	
	No	84	65.63	
Enough income to cover hemodialysis	Yes	6	4.69	
	No	122	95.31	
Coping Strategy	Staying at home	16	13	
	Minimizing sessions per week	56	46	
	Minimizing medications	52	43	
	Visiting traditional healers	2	2	
	Borrowing money	29	24	
	Support from neighbors and relatives and friends	79	65	
	Selling assets	47	39	
	Begging on the streets	8	7	
	NGO’s and associations provide additional support	11	9	

### Coping strategies

The primary coping mechanism utilized by most patients for the financial burden was support from relatives and neighbors (64.8%) (Table 5).

### Factors associated with cost of hemodialysis treatment

Linear regression was used to identify predictors of the cost of hemodialysis. Variables with p value less than 0.2 in the simple linear regression: type of institution, age, marital status, educational status, occupation, wealth status, type of vascular accesses, duration on hemodialysis, comorbidity status, anemia, CKDMBD, diabetes, treatment, place of purchase (medication), mode of transport for visiting health facility, and other facilities visited were considered for the final model. However, in the final model type of institution and the length of stay on hemodialysis were found to be independent predictors of the cost of hemodialysis (Table 6).

**Table 6:** Factors associated with the cost of hemodialysis at selected institution in Addis Ababa, Ethiopia, 2021 (*n* = 128)

Variable	β (Mean)(bivariable)	Adjusted β (Mean) (Multivariable)
**Type of Institution**		
Private	4518.415 (3908.87–5127.96)	4051.992 (2987.36–5116.62) *
Public	1	
**Age (years)**		
56–65	1261.94(-303.518–2827.39)	-310.69 (-1615.62 - 994.24)
18–35	1	
**Marital Status**		
Married	1185.05(207.54–2162.56)	621.89( -192.83 1436.54)
Unmarried	1	
**Educational Status**		
Primary Education	2156.63(-1075.19–5388.45)	-146.48( -2603.05–2310.09)
No education	1	
**Occupation**		
Unemployed	999.84 (2.14- 1997.55)	-132.11( -957.43 - 693.21)
Employed	1	
**Wealth status**		
High	2133.94 (961.62–3306.26)	-21.06 (-1044.79–1002.68)
Medium	1286.61 (100.25–2472.98)	-255.84 ( -1173.86–662.18)
Low	1	
**Vascular access**		
Catheter	3817.42 (1045.37–6589.48)	518.60 (-1595.68–2632.88)
Fistula	1605.52 (-683.79–3894.83)	111.95 (-1537.24–1761.16)
Graft	1	
**Duration hemodialysis (years)**		
< 1	4586.71 (3445.65–5727.78)	1479.09 (53.53–2904.65) *
1–3	2570.43 (1696.44–3444.41)	425.55 (-535.75 -1386.85)
> 3	1	
**Comorbidity status**		
Multiple Comorbidity	2150.91 (246.30–4055.51)	1999.17 (-2476.80 6475.13)
No Comorbidity	1	
**Anemia**		
Yes	1	
No	1388.99 (52.48–2725.49)	485.5478 (-712.28–1683.38)
**CKDMBD**		
Yes	1145.864 (111.71–2180.01)	487.29 (-286.72 - 1261.30)
No	1	
**Diabetes**		
Yes	1417.69 (107.16–2728.21)	258.69 (-691.22–1208.61)
No	1	
**Treatment**		
Multiple Medication	1582.38 (-460.77–3625.53)	-708.47 ( -4637.73–3220.79)
Single Medication	2420.36 (-596.54–5437.26)	-1152.17 (-5344.03–3039.70)
No medication	1	
**Source of medication**		
Private drug store	3668.57(2349.43- 4987.70)	-178.86 (-1361.13 -1003.42)
Both government and private drug store	909.09(2349.43–4987.70)	-599.92 (-1523.08 -323.24)
Government drug store	1	
**Type of transport**		
Private car	3018.06 (-493.69–6529.81)	134.96 (-2584.98–2854.9)
Ride	2223.49 (-1396.33–5843.33)	14.30 ( -2794.79–2823.39)
Public Transport	2356.90 (-930.97–5644.78)	-749.60 (-3354.60 -1855.40)
Walked	1	
**Visit other facilities**		
Yes	722.12 (-317.78–1762.01)	659.45 (-103.23 - 1422.14)
No	1	

*p value < 0.05

It was found that attending hemodialysis in a private facility increases the mean cost by 4051.99$ ± 535.804$ (2987.36–5116.62) as compared to attending treatment in public facilities. Similarly, patients who started hemodialysis treatment recently or more specifically less than a year were predisposed to spend 1479.09 $ ± 717.45$(53.53–2904.65) more than their counterparts (Table 6).

## Discussion

This study sought to assess direct and indirect cost of hemodialysis among end stage renal disease patients and associated factors among selected government and private institutions in Addis Ababa, Ethiopia. The study has revealed that the cost of hemodialysis was 7,739.17$ ±2,833.51$, with direct medical cost contributing 72.9% of the total cost. In addition, the majority of the patients reported that they could not to afford the treatment they received, many of them employing different coping mechanisms to cover their costs. The most reported coping mechanisms were support from friends and relatives, skipping hemodialysis sessions and skipping essential medications until the fund became available. Furthermore, the type of institution, private or public, was found to significantly predict the cost of hemodialysis, with more cost associated with visiting private facilities. There was also a significant association between the duration of hemodialysis and the cost of hemodialysis.

The cost of hemodialysis in the current study was comparably higher than a previous multicenter study in Ethiopia. The study, conducted in tertiary public hospitals of Addis Ababa and the Amhara region, found the total cost of hemodialysis to be4,466.59 $ [12]. However, in line withour study, the majority of the cost in this study is attributed to direct medical cost. The cost discrepancy between our study and the one conducted can be explained by several factors. The difference in the study period and the inclusion of participants from both private and public facilities in our study, as opposed to a previous study that only focused on government facilities, could account for our higher costs. Furthermore, the observed cost disparity between private and public facilities might contribute to this difference. Similarly, a report from Sudan has delineated a lower cost of hemodialysis, 6847$ [10]. This lower cost might be explained by the difference in the health system, including health insurance. However, similar to our study, a large proportion of the cost was attributed to direct medical costs. Different from the current study, a higher cost of hemodialysis was reported in Kenya, 16,845 $ [6]. Decreased household budget and increased health care expenditure in Kenya might explain this discrepancy.

Furthermore, other African countries have reported higher hemodialysis cost than the current study. As an illustration, the cost of hemodialysis in South Africa, and Tanzania was 31,993$ [8], and 27,440$ [9] respectively. The cost disparity between this study and ours might be explained by the different research designs, where they identified the cost from the healthcare provider perspective, and we identified the cost from the patient perspective. Although these studies uncovered a higher direct medical cost, they could better account for it as they conducted a cost analysis from the healthcare point of view, costing each process. Moreover, the cost of dialysis in the current study is lower than costs from upper-middle- income countries. For example, research done in Guangzhou, China and Malaysia identified the cost of hemodialysis to be 15,066 $ [14] and 9,253$ [15], respectively (14),(15). Regarding associated factors, the number of years on hemodialysis and the type of institution have shown a significant association with an increased cost of hemodialysis. Patients who started hemodialysis in less than a year were found to incur more costs than those who have been on hemodialysis for longer. The number of new laboratory tests, and medications at the beginning of the test might explain this finding; contrary to this, patients with longer duration might only have routine costs to bear. Besides, the cost of hemodialysis and other treatments are predominantly covered by out-of-pocket expenditure; newly diagnosed patients will incur more costs as they will not know treatment providers to cut costs. Moreover, price subsidization by private institutions for patients who have been there longer duration might also explain this scenario.

Finally, the significantly higher cost of hemodialysis among patients from private health institutions might be explained by the fee waiver being provided in the public health facilities of Addis Ababa.

The following limitation of the study cannot go unnoticed. For instance, this research was conducted in selected institutions in Addis Ababa, selected through subject matter expert consultation for the volume of patients they had. Therefore, the research does not represent all end stage renal disease patients in Addis Ababa and the larger Ethiopia. In addition, although sufficiently trained, we anticipated interviewer bias might exist due to the pre-existing relationship between our data collectors and our patients. Furthermore, as this study is conducted from the patient perspective, we expect over- estimation or underestimation of the cost by patients due to recall bias. Finally, we were unable to establish a causal link between the dependent and the independent variables because of the cross-sectional nature of the study design.

### Implication of study

This study highlights the significant cost implications of being an end stage renal disease patient on hemodialysis. It also provides insight into how patients cope with this significant disease burden. Some of these coping mechanisms predispose the patients to adverse health outcomes, as a significant portion of them skip hemodialysis treatment and other medication due to cost implications. These coping mechanisms have substantial implications for their survival and quality of life. This study will provide the perspective behind the fund-raising minivans parked on the streets to keep some patients in the hospital. This research might provide government officials and non-governmental organizations with insights into the conditions these patients face and encourage them to implement programs that will support them. Further study is required from the healthcare provider perspective to identify which cost drivers be it labor, reagents, and infrastructure inflate direct medical costs for these patients, and we have not found any such literatures on this during the preparation of this paper.

## Conclusion

Our study uncovered a previously under reported higher average cost associated with end stage renal disease patients undergoing hemodialysis in the selected institutions. The first cost-driving factor is the type of institution the patient receives care, whether public or private. At the same time, the second driving factor is newly joining this treatment which had higher cost as compared with more experienced patients. It also provides insight into how patients cope with this significant disease burden. Some of these coping mechanisms predispose the patients to adverse health outcomes, as a significant portion of them skip hemodialysis treatment and other medication due to cost implications. These coping mechanisms have substantial implications for their survival and quality of life. This study will provide the perspective behind the fundraising minivans parked on the streets to keep some patients in the hospital. This research might provide government officials and non-governmental organizations with insights into the conditions these patients face and encourage them to implement programs that will support them. Further studies are required from the healthcare provider perspective to identify which cost drivers be it labor, reagents, and infrastructure inflate direct medical costs for these patients, and we have not found any such literature on this during the preparation of this paper.

## Data Availability

Dataset used will be available upon request from the corresponding author.

## Abbreviations

CKD: Chronic Kidney Disease
CKDMBD: Chronic kidney disease mineral bone disease
CVD: Cardiovascular disease
ESRD: End Stage Renal Disease
ETB: Ethiopian Birr
GDP: Gross domestic product
km: Kilometre
NGO: Nongovernmental organization
SD: Standard Deviation
STATA: Statistical Software for Data Science
USD: United States Dollar
VIF: Variance Inflation Factor
